# In the Shadows of Patients with Upper Gastrointestinal Cancer: An Interview Study with Next of Kin about Their Experiences Participating in Surgical Cancer Care

**DOI:** 10.1177/1054773820940873

**Published:** 2020-07-10

**Authors:** Farzana Ibrahim, Carina Wennerholm, Per Sandström, Anna Lindhoff Larsson, Bergthor Björnsson, Jenny Drott

**Affiliations:** 1 Linköping University, Linköping, Sweden

**Keywords:** next of kin, cancer care, participation, involvement, qualitative research

## Abstract

The study aimed to explore the experiences of participation among the next of kin of patients who had surgery for upper abdominal tumours. This study had a qualitative research design and data were analysed by thematic analysis. Eleven qualitative interviews were conducted with next of kin to patients who had surgery for liver, bile duct or pancreatic malignancy. The following themes emerged: *from the shadows to an important role* and *an inviting and inhibiting environment for participation.* Next of kin were a central part of their loved one’s care but often in the shadows. The next of kin described how they were not always invited and often they had to struggle to get involved. Next of kin often have a major psychosocial role to supporting the patient during and after discharge, and therefore need to be invited and involved in the whole care process.

## Introduction

Being diagnosed with cancer in the liver, bile ducts and pancreas is a traumatic experience, not only for the patient but also for the next of kin. The next of kin refers to a person close to the patient with a cancer diagnosis in the liver, bile ducts and pancreas. During the waiting time for surgery, both patients and the next of kin expect successful results. Patients with cancer in the liver, bile ducts and pancreas often have a major surgical procedure to go through and a long period of recovery. It is common with a short hospital stay and it often includes a long recovery period at home. With short hospital stay the next of kin often have a central role. Despite the importance of next of kin, health care professionals focus to a great extent on the patient’s needs. Many times, next of kin have greater informal and psychosocial needs than patients do because they try to be supportive while taking responsibility for everything else. For this reason, it is important that professionals create a supportive consultancy environment that encourages interaction with next of kin (Madsen et al., 2019; Reinares et al., 2016).

Cancer affects the whole family in various ways, and crisis reactions, loneliness, emotional distress and anxiety are common (Gray et al., 2019). The next of kin lives with the risk of losing their loved ones after the cancer diagnosis. Feelings of stress and suffering are common and are often endured in silence (Partanen et al., 2018; Peikert et al., 2018).

Participation in care is one of the cornerstones of high-quality care. Patient participation is a concept closely related to patient safety (Longtin et al., 2010). Earlier research has shown that patient participation is associated with improved recovery, rehabilitation and treatment outcomes (Castro et al., 2016). The importance of family members as supporters of patients with cancer is recognised. Healthcare professionals often appreciate family members and next of kin. Sometimes professionals see next of kin as beneficial in the care situation. Next of kin need information, encouragement and support to be involved in their loved ones’ care. The participation of next of kin may shorten hospital stays and enhance coping at discharge and at home. Healthcare professionals have an important role to play in improving the well-being of patients and relatives. It is important that healthcare professionals take the time to truly understand relatives’ needs and worries. Showing empathy can prevent suffering, reduce anxiety and stress. Additionally, healthcare professionals should be honest and convey the truth about health status to build trusting relationships (Berry et al., 2017). Existing scientific evidence regarding the participation of next of kin in cancer care is sparse, and only a few studies on this topic have been published during the last three decades (Partanen et al., 2018).

Accordingly, research is lacking about patients undergoing surgery for upper abdominal cancers concerning their next-of-kin experiences of participation in surgical care.

Previous studies about patient participation in surgery cancer care for this patient group, highlight the patients’ needs for person-centred information (Ibrahim et al., 2019; Larnebratt et al., 2019). Knowledge about next-of-kin experiences of involvement may improve tailored care for patients with surgery for upper abdominal cancers. The present study aimed to explore experiences of participation among next of kin of patients who had surgery for upper abdominal tumours.

## Methods

This study had a qualitative research design and aimed to shed light on next-of-kin experiences and thoughts in surgical cancer care. In line with the Declaration of Helsinki (WMA, 2013), next of kin were informed about the study orally and in writing, and written informed consent was obtained. This study was approved by the Research Ethics Committee in Linköping, Sweden (No. 2016/276-31). The data collection and analysis followed the COREQ checklist.

### Sample and Setting

Next of kin to patients who had surgery for liver, bile duct or pancreatic malignancy were recruited from a specialist surgical clinic in a university hospital in southern Sweden between the summer of 2018 to the beginning of 2019. Eleven agreed to participate in the study and were included. The sampling strategy was purposeful, and we aimed for a variation of different characteristics (e.g. sex, age, relations). Table 1 summarises the interviewees’ characteristics at the time of the interview.

**Table 1.** Characteristics of the Next of Kin.

Next of kin	Sex	Age	Relation	Patients type of malignancy
1.	Female	68	Wife	Pancreatic
2.	Female	73	Wife	Pancreatic
3.	Female	27	Daughter	Pancreatic
4.	Male	63	Husband	Liver
5.	Female	34	Daughter	Pancreatic
6.	Female	66	Wife	Liver
7.	Female	60	Wife	Liver and bile duct
8.	Male	48	Son	Pancreatic
9.	Female	76	Wife	Liver
10.	Male	81	Husband	Pancreatic
11.	Male	42	Son	Liver

### Procedure

An interview guide for participation that included open-ended questions was used. The interview guide was pilot tested, and no amendments were made. The data from the pilot interview was included in the data analysis. Since the surgical clinic had patients from different areas in southern Sweden and the next of kin lived far away from the hospital, interviews were conducted both by phone and face to face. The interviews were conducted by the first and last authors between 1 to 3 weeks postoperatively. Both interviewers have surgical specialist clinical experience. The next of kin were contacted and informed about the study orally when they contacted the patients in the surgical clinic or informed by telephone. The date of the interview meeting was decided by the next of kin. They also decided whether a face-to-face meeting or an interview by telephone should be used. Before the interview began, general conversation with next of kin started the interview meeting. All interviews started with a question on next-of-kin experiences regarding their participation in relation to their partners’ and loved ones’ surgical cancer care. Interview areas included follow-up questions and clarifications about participation and experiences of involvement. Looping and probing questions were used continuously during the interviews, according to existing qualitative literature (Patton, 2015). The interviews lasted between 13 and 67 min (median 36 min). The 13-min interview was conducted with a next of kin (No: 11) by telephone, it was short but added important information according to the aim of the study. He lived far away and has small children at home. All the interviews were transcribed verbatim.

### Data Analysis

The data analysis started during the interviews and the data collection. A thematic approach was used according to the analysis (Braun & Clarke, 2006). The interviews were transcribed and analysed by the authors. All the transcribed text was read and re-read to facilitate familiarisation and comprehension of the data. Initial codes were generated and were identified according to the study aim. All codes relevant to the aim were incorporated and defined the themes. The thematic analysis followed: the familiarisation with the data, generation of initial codes, search for themes, review of themes, definition and naming of themes and production of the report (Braun & Clarke, 2006). Transcripts were analysed and interpreted independently, and the findings were compared among the researchers. Discussions in the research group about the findings were carried out in order to increase the trustworthiness. The criteria by Lincoln and Guba for establishing trustworthiness in qualitative research were used; credibility, dependability, confirmability and transferability. The sampling strategy was purposeful, and we aimed for a maximum variation of different next of kin and experiences about participation to enhance credibility. Discussions among the research team about the results were carried out to reach consensus and trustworthiness (dependability) of the findings. The quotations in the findings illustrated the next of kins real words (confirmability). To make these findings transferable to other surgical cancer contexts, descriptive information about the included next of kin is presented in Table 1 and makes it possible for the reader to evaluate the findings to other settings (Lincoln & Guba, 1985; Nowell et al., 2017).

## Results

The two main themes that emerged were *from the shadows to an important role* and *an inviting and inhibiting environment for participation.*

### From the Shadows to an Important Role

The experience of next of kin was that they were a central part of their loved one’s care but were often in the shadows. The next of kin described how they struggle for their loved ones and participated in their care and care planning.

*I have shielded myself, because when I was told it was cancer, I was so shocked and frightened. We have a family we spend time with, and there the spouse also had cancer. So, we’ve talked about it, how it was to stand close and somehow, I shut myself off, because I couldn’t really cope with it, that he has cancer and he may die. It sounds so weird, but I shielded myself in some way, I went into my own little world (Next of kin 7).*

A lack of information about postoperative complications, surgery or treatment and discharge planning resulted in dissatisfaction and anxiety among the next of kin. The consequence was that next of kin felt stressed and were unprepared for what happened. Written information caused great concern for the next of kin.

*We got home a sick leave document where it said that there was a risk that it was palliative care. I was totally cold and shielded myself from the surroundings. I do not know why they wrote so, but it was of course before they knew if there was metastasis. I was quite anguished and turned off mentally. Later, they started to talk about the tumour and the intention to remove it, and it should probably go well (Next of kin 6).*

The next of kin have a supportive need for information after they received the written information, and they wanted explanations and know more about what to expect. They also wanted to do their best to protect and support the patients and wanted to participate and contribute to the decision-making process. Next of kin often tried to be positive and organised all practical activities in the normal life, but they hide their own feelings in the shadow.

Patients who had been discharged from the clinic to other smaller hospitals for recovery after a few days sometimes lacked discharge calls and a treatment plan discussion with a doctor. The next of kin to the patients had many questions that they felt were not answered when they had their relatives at home.

*It was very poor information, no information at all really in connection to the discharge from the hospital. . . There was no discharge information; we really miss the information (Next of kin 9).*

Several next of kin also emphasised the importance of information, for example, about complications that occurred during the care period. They did not receive enough information and felt like they were in the shadows of the patients and were not truly invited to the rounds or conversations during the hospital care period. When information was provided, it was usually given at the discretion of health care professionals and, at times, decided upon by the same. However, when it was time to leave the hospital, the health care professionals often expected that next of kin take a great responsibility of the patients’ care at home.

*. . .Yes, there are a lot of questions. . . It would have been good if you could get deeper discussions about the expected situation, lots of such questions that have been solved by your own. . . Like this with the food. . . The dietician was involved and connected, but you might have been able to get some more information about suitable food. I have the responsibility for the cooking now, I mean, I’m not so young either. . . We are both old. . . and lacked the information about the appropriate food (Next of kin 10).*

Several next of kin missed the conversations with the professionals. In most cases, they were informed by their beloved sick family member and not via direct dialogue with health care professionals.

*It would have been good if I had had the opportunity to participate more actively in conversations that my wife had with doctors and nurses about her situation. . . But. . . If I had been opinionated, I would have been able to demand a conversation. . . You may be taking enough initiatives yourself, but it is quite a lot that you must think about anyway (Next of kin 4).*

Several of the interviewees showed a desire to be more involved in the decisions made regarding their family member who had surgery for cancer.

*. . . I visited my wife in the hospital and met one of the surgeons and had some conversations about the surgery. . . It is very nice to talk to the person who has performed the surgery, to get first-hand information (Next of kin 10).*

### An Inviting and Inhibiting Environment for Participation

An inviting atmosphere stimulated by the health professionals increased feelings of participation, reduced fears and stress.

*It could even be that assistant nurses ask me. . .. I’ve been in attendance every day and know a lot. The staff were very friendly and helpful. But just like this ridiculous stuff. . . like when he could pee himself on the toilet after surgery, there was someone who came in and hugged him on the toilet and then hugged me. . .well, it sounds a little ridiculous (Next of kin 1).*

Some next of kin experienced that they felt more involved when the staff involved them also in the patients’ privacy care. The constant discussions of current care planning and the fact that the next of kin felt free to call the clinic with questions or concerns entailed a feeling of security.

*I felt very involved, because they told me. . . They told me how it would go and that we had to call, I knew I could call if I wanted to ask, and I had to find out much about the surgery. . . I’m very satisfied. . . (Next of kin 8).*

Several relatives experienced a lack of medical conversations. They wanted the surgeon to come and tell them about the surgical procedure. Their loved ones did not remember what was said during the medical rounds and could not reproduce the information. Several relatives wanted the surgeon to call them and communicate how the surgery passed, but no one did. This dialogue was missed because they had questions they wanted to be answered. They also wanted to be invited to the rounds in the hospital and participate, but they were never invited to participate.

*When he was moving from the hospital to another hospital, I did not feel safe. Neither did he. We wanted to have information; it went too fast. No one said anything; then suddenly, the move would take place the next day. It must be possible to have a better plan before discharge to another hospital (Next of kin 2).*

*My husband was in bad health one night, and I received an SMS from him that he would be moved to the intensive care unit. But there was no staff who called me; I was 300 km away and became worried. Then, I called the hospital and talked to a staff member who said that she did not know anything. I felt it negligent. I was far away and knew nothing about what happened, and no one had called me. You are very vulnerable and exposed as a next of kin (Next of kin 6).*

It was also next of kin who felt that they had to take a large responsibility for assessment after discharge, which felt heavy and stressful. There was no problem in emptying the drainage and completing other tasks, but it was a hassle to make assessments when one had not experienced the health care for which they took responsibility.

**Figure 1.**
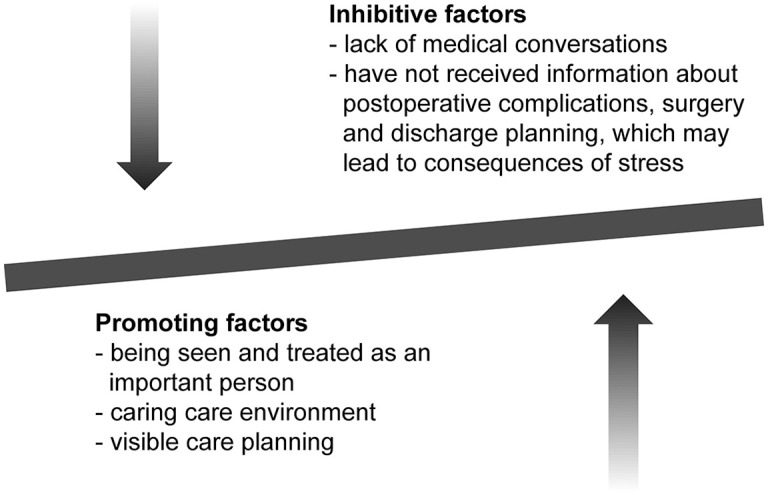
Promoting and inhibitive factors for next-of-kin participation.

The findings from the interviews show both promoting and inhibiting factors, as summarised in Figure 1.

## Discussion

The main interpretation of our results shows that there are no implemented clinical routines to include next of kin in the surgery cancer care process. The main results were two themes: *From the shadows to an important role* and *an inviting and inhibiting environment for participation.* The provision of support to the next of kin is important in upper gastrointestinal cancer surgery care. An inviting atmosphere can reduce the stress and distress experienced by the next of kin. An inviting and inhibiting nursing environment may improve the care quality. To our knowledge, this interview study is the first to focus on next-of-kin experiences of participation when their loved ones have had surgery for liver, bile duct or pancreatic cancer. The next of kin perspectives and experiences from this study aimed to improve the clinical care. When a family member is diagnosed with cancer, the whole family is affected by anxiety and ill mental health. Thoughts about different treatments and the future are common. Often, relatives are looking for information and knowledge to take advantage of the best treatments and cures in the best way. A patient’s experience with a cancer diagnosis and its impact can lead to major psychological stress for the family members who are close to the patient (Caruso et al., 2017).

The results of this study show that it is important for the next of kin to be involved in the whole care process. These results are similar to those of previous studies in which the importance of relatives in the patient’s care is emphasised (Leino-Kilpi et al., 2016; Madsen et al., 2019; Otutaha et al., 2019). Family members are often a great support for patients, and they often undertake many responsibilities during the disease period. It is common for relatives to feel unprepared for such major responsibilities and therefore need the help and support of health professionals. Next of kin experienced information from health professionals as a source of support and security. For this reason, it is important that next of kin, together with the patient, are involved in the patient’s care (Kwon et al., 2018; Leino-Kilpi et al., 2016).

Honest communication is of great importance (Otutaha et al., 2019; Yang et al., 2018). Environments are perceived as very positive when relatives can share their experiences, obtain information and knowledge from healthcare professionals and be involved in patient care. This kind of contact is highly appreciated and leads to patients feeling safer when they know that their relatives have knowledge and can cope with different situations around healthcare, even at home (Koop & Strang, 2003; Norinder et al., 2017).

One of the findings of this study was the theme *from the shadows to an important role*. The next of kin indicated that they felt like a shadow, or that they were included in one way but without the involvement of health professionals. This experience could lead to a sense of alienation and loneliness, and they felt invisible. To relate these findings to those of other studies and to clinical success factors, for example, studies in paediatric cancer care and family-centred care have shown that advice related to psychosocial aspects and to the conveying of hope was important for family members. Information, communication and engagement should be addressed to support psychosocial needs (Lovgren et al., 2016; Mittal, 2014), and family relationship may have a major role (Reblin et al., 2019).

Previous studies have shown that health care professionals should carefully select the appropriate time to inform patients of their diagnosis and actively meet patients’ needs for person-centred information (Hakansson Eklund et al., 2019; Ibrahim et al., 2019; Larnebratt et al., 2019; Schildmann et al., 2013; Tariman et al., 2010; Yang et al., 2018). The findings of this study indicated that the next of kin felt mentally blocked, went into their own world and could not cope with the situation. This psychological defence reaction highlights the importance of carefully selecting the appropriate time to inform next of kin. Based on the stressful situation of cancer in the family, professionals need to actively and carefully tailor their provision of information to the needs of family members.

A central issue is why health professionals often ‘forget’ next of kin in the patient’s care process and what they can do to improve contact with next of kin and involve them more in the care. In a study by Laidsaar-Powell et al. (2018), the results show that next of kin should be followed regularly, be offered emotional support and be informed about their sick family member’s health status, treatments and care planning by health care professionals during oncological treatment. Next of kin often need support and guidance in a greater psychosocial need than the patient’s self. Next of kin often have a supportive and caring role and taking all responsibility for the house and family. It is important that professionals create a supportive environment during the hospital stay and offer support after discharge (Madsen et al., 2019; Reinares et al., 2016). The findings of this study also indicated that next of kin have not received enough information about expected care time or postoperative complications, which can lead to stress and anxiety. It is important that next of kin receive knowledge and information (Kynoch et al., 2016) and that information and interventions are adapted to the specific situation (Hu et al., 2019; Moore et al., 2018). Patients and their next of kin can be supported together to addressing needs and issues of fear (la Cour., 2016).

Nurse’s role is very important especially in this cohort of patients and their families. Nursing is dynamic and patient centered. Supportive and educative components are central in the nursing role, and nurses often adapt to situations if the patients and families need nursing support. Finally, this study demonstrates the importance of information to the next of kin. The findings show that next of kin who received information experienced a feeling of security and safety. Family members who did not feel seen and noticed had feelings of anxiety and fear, and they felt that they were like a shadow. Next of kin often play a major role in caring for patients, and it is important that they are properly informed to cope with the situation.

### Strength and Limitations

In the interpretation of the findings, methodological limitations must be considered. Initial codes were generated and were identified according to the study aim. All codes relevant to the aim were incorporated and defined the themes in the study. The process of searching for themes due to the method (Braun & Clarke, 2006) may be a critical part of the interpretation. Discussions in the research group about the themes and the findings were carried out in order to increase the trustworthiness. The procedure to conduct the analysis aims to meet the trustworthiness criteria by Lincoln and Guba. The sampling strategy was purposeful, but we aimed for a variation of different characteristics of next of kin to enhance credibility. Next of kin were only recruited from one hospital, which might be a limitation. On the other hand, five of the patients to the next of kin had a very short hospital stay in the university hospital and moved to a small local hospital after a few days postoperatively. A strength was that interviews were carried out between 1 to 3 weeks postoperatively, and next of kin remember a lot of details regarding their specific surgical care situation. The sample size in the study was quite small, but the data were sufficiently rich to fulfil the study aim. The experiences of the included next of kin and their relations in the study may differ, which is interpreted as a strength of the study.

### Clinical Implications

When a family member is diagnosed with liver, bile duct or pancreatic cancer, the whole family is affected by anxiety and psychological stress. The provision of nursing support to the next of kin is important in upper gastrointestinal cancer surgery care. A nurse’s responsibility is to support both patients and their family members to maintain health. An inviting atmosphere through the whole cancer surgery care process can reduce the stress and distress experienced by the next of kin. The findings show that the next of kin who received information when they needed, experienced an increased feeling of security and safety. Nursing support should be dynamic and holistic, and it is important that next of kin are supported and properly informed to cope with the situation over time. Nurses often play a major role in caring for patients, and the implication for nursing is to involve next of kin more actively during the care process. The findings show that next of kin who been seen of the health professionals, and received information experienced a feeling of safety. Psychological defence reaction highlights the importance of carefully selecting the appropriate time to inform next of kin. An inviting atmosphere can reduce the stress and distress experienced by the next of kin, and it is common for next of kin to feel unprepared for major responsibilities of patient’s health. Further research will need to identify strategies to support next of kin in clinical practice and ensure that these strategies achieve their intended goals of promoting factors to improve participation. Further research will also focus on social support and family centred care.

## Conclusion

In conclusion, we found that next of kin play a major role in supporting the patient and therefore need to be involved in care and involved in the whole care process.

The main interpretation of our results shows that there are no implemented clinical routines to include next of kin in the surgery cancer care process. Supportive and educative components are central in the nursing role, and nurses often adapt to situations if the patients and families need nursing support. Psychosocial support for cancer related situations often either focuses on patients or next of kin. Although it is also important how next of kin and their loved ones can be supported together in the surgical cancer care.
